# The Effects of Community Home Visit and Peer Group Nutrition Intervention Delivery Platforms on Nutrition Outcomes in Low and Middle-Income Countries: A Systematic Review and Meta-Analysis

**DOI:** 10.3390/nu12020440

**Published:** 2020-02-10

**Authors:** Amynah Janmohamed, Nazia Sohani, Zohra S Lassi, Zulfiqar A Bhutta

**Affiliations:** 1 Centre for Global Child Health, The Hospital for Sick Children, Toronto, ON M5G 2L3, Canada; janmohamedamynah@gmail.com (A.J.); naziasohani@gmail.com (N.S.); 2 Robinson Research Institute, University of Adelaide, Adelaide SA 5006, Australia; zohra.lassi@adelaide.edu.au

**Keywords:** nutrition, intervention, delivery, systematic review, low and middle-income country

## Abstract

Weak delivery systems reduce the potential of evidence-supported interventions to improve nutrition. We synthesized the evidence for the effectiveness of nutrition-specific intervention delivery platforms for improving nutrition outcomes in low and middle-income countries (LMIC). A systematic literature search for studies published from 1997 to June 2018 resulted in the inclusion of 83 randomized controlled trials (RCTs), quasi-randomized, and controlled before–after studies across a variety of delivery platforms. In this paper, we report on meta-analysed outcomes for community health worker (CHW) home visits and mother/peer group delivery platforms. Compared to care as usual, CHW home visits increased early initiation of breastfeeding (EIBF) (OR: 1.50; 95% CI: 1.12, 1.99; *n* = 10 RCTs) and exclusive breastfeeding (EBF) (OR: 4.42; 95% CI: 2.28, 8.56; *n* = 9 RCTs) and mother/peer groups were effective for improving children’s minimum dietary diversity (OR: 2.34; 95% CI: 1.17, 4.70; *n* = 4) and minimum meal frequency (OR: 2.31; 95% CI: 1.61, 3.31; *n* = 3). Pooled estimates from studies using both home visit and group platforms showed positive results for EIBF (OR: 2.13; 95% CI: 1.12, 4.05; *n* = 9), EBF (OR: 2.43; 95% CI: 1.70, 3.46; *n* = 12), and < 5 wasting (OR: 0.77; 95% CI: 0.67, 0.89; *n* = 4). Our findings underscore the importance of interpersonal community platforms for improving infant and young child feeding practices and children’s nutritional status in LMICs.

## 1. Introduction

Poor nutrition in the early years of life poses substantial, and potentially lifelong, costs to health, development, and socioeconomic well-being [1,2]. Preventing the adverse effects of undernutrition requires essential nutrition actions during the critical first 1000 days of life [3]. Despite evidence-supported interventions to improve maternal and child nutrition [4], their potential gains are often thwarted by weak delivery systems preventing maximum population reach and impact [5,6].

In most countries, a variety of intervention delivery platforms, or channels, are used to reduce access barriers to essential services. Notable examples are trained community health workers (CHWs) and mother/peer groups for health and nutrition promotion [7,8,9]. Though evidence supports CHW home visits and women’s groups for improving maternal and newborn health in low and middle-income countries (LMICs) [10,11,12], evidence from high-quality studies involving such community platforms for delivering nutrition-specific interventions is limited across diverse populations [5]. This is partly due to limited knowledge surrounding how best to integrate nutrition-focused interventions into these routine activities at the country level [13].

Emerging evidence indicates that advancements in nutrition require nutrition-sensitive programmes that enhance the coverage and effectiveness of direct nutrition interventions [14]. Cash transfers requiring compliance with specific health/nutrition behaviours are commonly used in social protection/safety net programmes in LMICs [15,16]. Though evidence suggests they are effective for poverty reduction [14,17,18], there is limited high-quality evidence on the effectiveness of conditional cash transfers (CCTs) for improving nutrition-specific outcomes among women and children [19]. Mobile health (mHealth) technologies (e.g., SMS messaging) are increasingly being used in global nutrition programming, yielding potentially cross-cutting benefits through linkages with community and financial platforms [20]. However, knowledge gaps remain surrounding the effectiveness of mHealth platforms for delivering nutrition-specific interventions [21,22].

We reviewed the available evidence to assess the effects of community-, financial- and technology-based nutrition intervention delivery platforms for improving nutrition outcomes in LMICs. In this paper, we report on meta-analysed outcomes for community health worker (CHW)/lay counselor home visits and mother/peer groups, which emerged as the most widely used community-based platforms for delivering nutrition-specific interventions in our review. While the multiplicity of health delivery platforms across LMICs is acknowledged, our review focused only on those delivering a nutrition-specific intervention or that integrated a direct nutrition component.

## 2. Materials and Methods 

### 2.1. Types of Studies 

We conducted the systematic review and meta-analysis following the Preferred Reporting Items for Systematic Reviews and Meta-Analyses (PRISMA) guidelines [23] and according to the study protocol [24]. We searched the Cochrane Central Register of Controlled Trials, Embase, MEDLINE, Scopus, and Web of Science for English-language studies published from 1997 to June 2018 using Medical Subject Heading (MeSH) terms and keywords (MEDLINE search strategy included in Table S1). In addition, ClinicalTrials.gov and the WHO International Clinical Trials Registry Platform were searched for ongoing trials and we examined the grey literature for relevant studies. Studies were included if they: (i) occurred in a LMIC, as per the World Bank [25] definition at the time of publication; (ii) utilized a community, financial and/or technology platform for delivering a nutrition-specific intervention; (iii) utilized a randomized, cluster-randomized, quasi-randomized, stepped-wedge, controlled before-after, or interrupted time series design with a control group; and (iv) examined a relevant outcome. Excluded studies did not include a control group.

### 2.2. Outcomes 

The primary study outcomes were: early initiation of breastfeeding (EIBF) (breastfeeding within one hour of birth), exclusive breastfeeding (EBF) (up to 6 months), minimum dietary diversity (MDD) and minimum meal frequency (MMF) (based on WHO [26] guidelines), compliance with micronutrient supplementation, anthropometric measures (height/length, weight, body mass index, stunting, underweight, wasting), hemoglobin/anemia, and micronutrient status. No secondary outcomes were assessed. Duration of exposure and timing of outcome measurement were not restrictions for exclusion.

### 2.3. Search Methods and Data Extraction 

Two reviewers screened titles and abstracts to identify potentially eligible studies using Covidence (Veritas Health Innovation, Melbourne, Australia). Full texts of selected articles were reviewed independently by two reviewers for eligibility based on predefined inclusion criteria, with discrepancies resolved by discussion. Double data extraction using standard forms was conducted for studies that met the inclusion criteria. Extracted data consisted of: (i) author, publication year, country, area; (ii) study design; (iii) study population characteristics; (iv) type of delivery platform; (v) description and duration of intervention(s); (vi) control group description; (vii) outcome measurement(s); and (viii) effect measure(s). Reference lists from included studies and relevant systematic reviews were also examined.

### 2.4. Statistical Analysis 

Pooled estimates of outcome measures were calculated using random-effects models (inverse-variance method) in RevMan 5.3 (Cochrane Collaboration). Meta-analyses were conducted based on outcome and type of delivery platform and, when possible, were performed separately for randomized and non-randomized studies. Pooled estimates are presented as odds ratios (OR) with 95% confidence intervals (CI). Where necessary, data conversions were made using available study data. For study outcomes measured at multiple time points (e.g., anthropometry), we selected similar measurements across studies based on what we considered to be the most clinically relevant follow-up period for the particular outcome. Statistical heterogeneity for included studies was assessed by visually examining forest plots and quantitatively using Tau^2^, I^2^, and significance of the chi-squared test. Sensitivity analyses were performed based on removing studies with outlying effect sizes.

### 2.5. Quality Assessment

Two reviewers independently assessed the risk of bias for all included studies using the Cochrane risk-of-bias tool [27] and the Cochrane Effective Practice and Organisation of Care (EPOC) guidelines [28] for randomized and non-randomized studies, respectively. As the blinding of study participants and intervention providers is relatively uncommon in behavioural intervention trials, we concluded that non-blinding posed a low risk of bias for outcomes we considered to be minimally affected by a lack of blinding. We used the GRADE method [29] to evaluate the overall quality of evidence and produce a summary of findings table for each meta-analysed outcome.

## 3. Results

### 3.1. Search Results

**Figure 1:**
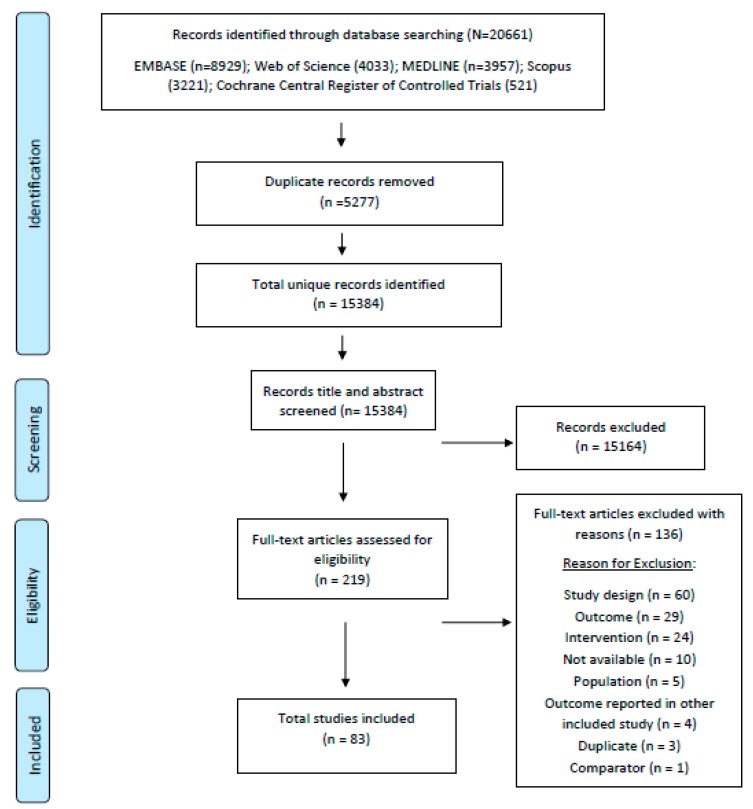
PRISMA diagram.

We identified a total of 20,661 records in the search, of which 5277 were duplicates, and 15,164 were excluded at the title/abstract screening stage. We reviewed 219 full-text articles for eligibility and a total of 83 studies were included in the full review (Figure 1). Table S2 summarizes the key characteristics of the CHW/lay counselor home visit and mother/peer group studies, which are the focus of this paper. These studies utilized one or both of these platforms to deliver nutrition education/behaviour change communication to mothers/caregivers of young children.

### 3.2. CHW/Peer Counselor Home Visit Platform 

**Figure 2:**
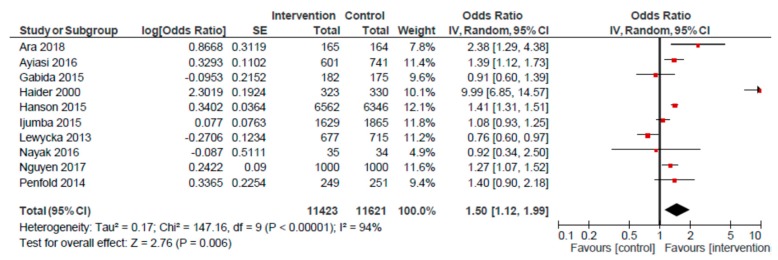
Forest plot of the meta-analysis of early initiation of breastfeeding for home visits (randomized studies).

For the CHW/peer counselor home visit platform, we conducted meta-analyses for four outcomes: EIBF, EBF, <5 stunting and < 5 underweight (Table 1). The pooled estimate for EIBF based on 10 RCTs showed a positive effect (OR: 1.50; 95% CI: 1.12, 1.99) for home visits, compared to usual care (Figure 2). A smaller, though positive, effect was observed when excluding the Haider et al. study from the analysis (OR: 1.20; 95% CI: 1.02, 1.41). A pooled analysis of non-randomized studies (*n* = 4) also revealed a higher likelihood of EIBF among mothers receiving home visits, as compared to those receiving usual care (OR: 1.80; 95% CI: 1.17, 2.78). 

**Figure 3:**
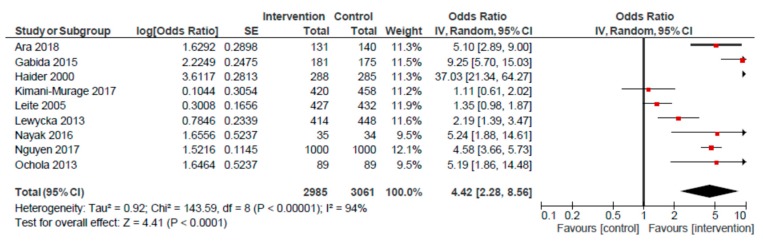
Forest plot of the meta-analysis of exclusive breastfeeding for home visits (randomized studies).

An analysis of nine RCTs examining the association between the home visit platform and EBF produced an overall effect of 4.42 (95% CI: 2.28, 8.56) (Figure 3). When removing the Haider et al. study, the odds of EBF remained significantly higher among mothers receiving home visits, compared to usual care (OR: 3.33; 95% CI: 1.94, 5.72). A pooled analysis of three non-randomized studies also revealed a positive effect (OR: 4.57; 95% CI: 1.36, 15.36) on EBF for mothers receiving home visits, compared to those receiving usual care. 

Meta-analyses revealed no significant impact of the home visit platform, compared to usual care, on < 5 stunting (OR: 2.12; 95% CI: 0.46, 9.74; *n* = 3) and a nearly significant 7% decrease in the likelihood of < 5 underweight (OR: 0.93; 95% CI: 0.82, 1.06; *n* = 4).

### 3.3. Mother/Peer Group Platform

**Figure 4:**
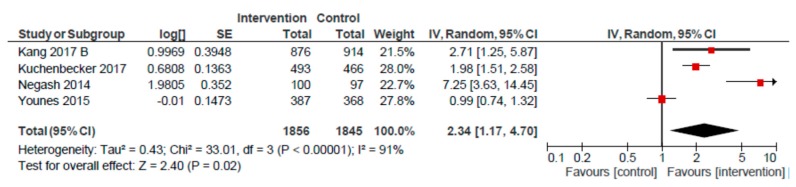
Forest plot of the meta-analysis of child minimum dietary diversity for mother/peer groups.

**Figure 5:**
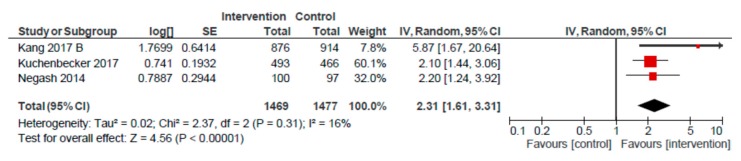
Forest plot of the meta-analysis of child minimum meal frequency for mother/peer groups.

For the mother/peer group platform, we conducted meta-analyses for seven outcomes: EIBF, EBF, MDD, MMF, and < 5 stunting, underweight and wasting (Table 2). Pooled estimates were non-significant for EIBF (OR: 1.10; 95% CI: 0.92, 1.32; *n* = 5), and EBF (OR: 1.78; 95% CI: 0.93, 3.41; *n* = 4), as compared to usual care. A pooled analysis of four studies revealed a higher likelihood of MDD (OR: 2.34; 95% CI: 1.17, 4.70) (Figure 4). However, removing the Negash et al. study resulted in a lower, and non-significant, effect on MDD (OR: 1.64; 95% CI: 0.92, 2.93) for children of mothers participating in group sessions, as compared to usual care. A similarly positive meta-analysed effect was observed for MMF (OR: 2.31; 95% CI: 1.61, 3.31, *n* = 3) (Figure 5). Removing the Kang et al. study reduced the likelihood of MMF to 2.13 (95% CI: 1.55, 2.92) for the mother/peer group platform, but remained significantly higher, as compared to usual care.

In a pooled analysis of three studies that examined the effects of the group platform on <5 stunting, underweight, and wasting, compared to care as usual, there was a nearly significant 15% reduction in the odds of stunting (OR: 0.85; 95% CI: 0.68, 1.07) and a 24% reduction in the odds of underweight (OR: 0.76; 95% CI: 0.53, 1.07). For child wasting, the observed effect was 0.86 (95% CI: 0.65, 1.14).

### 3.4. Combined Home Visit and Group Platforms

**Figure 6:**
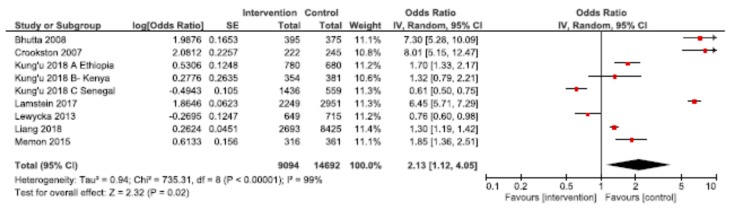
Forest plot of the meta-analysis of early initiation of breastfeeding for home visits and mother/peer groups.

**Figure 7:**
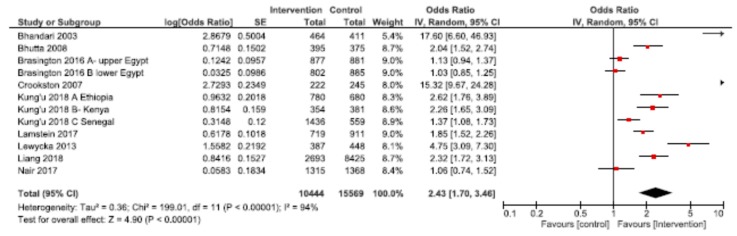
Forest plot of the meta-analysis of exclusive breastfeeding for home visits and mother/peer groups.

Through examining studies that used both home visit and group platforms for delivery of nutrition interventions, we explored the synergistic benefits of using both modalities for improving nutrition outcomes. Meta-analyses were conducted for EIBF, EBF and stunting underweight and wasting (Table 3). Pooled results showed a significantly higher likelihood of EIBF (OR: 2.13; 95% CI: 1.12, 4.05, *n* = 9 among mothers receiving home visits and participating in group sessions, compared to usual care (Figure 6). Excluding the Bhutta et al., Crookston et al., and Lamstein et al. studies reduced the likelihood of EIBF to a non-significant 1.15 (95% CI: 0.82, 1.62). For EBF, the observed effect was 2.43 (95% CI: 1.70, 3.46) across 12 studies (Figure 7). Removing the Bhandari et al. and Crookston et al. studies revealed a lower, though still significantly higher than in usual care, likelihood of EBF among mothers benefiting from home visits and group sessions (OR: 1.79; 95% CI: 1.38, 2.32).

**Figure 8:**
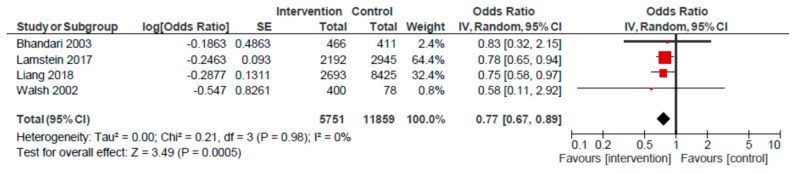
Forest plot of the meta-analysis of child wasting for home visits and mother/peer groups.

The impact of the home visit and group session platform on stunting among children < 5 years was 0.97 (95% CI: 0.72, 1.30) across a pooled analysis of eight studies. The impact on < 5 underweight was 0.84 (95% CI: 0.61, 1.14; *n* = 6). A 23% reduction in the odds of < 5 wasting (OR: 0.77; 95% CI: 0.67, 0.89) was observed across four studies that used the home visit and group platform, compared to usual care (Figure 8).

## 4. Discussion

In this paper, we have summarized the available evidence for the effectiveness of CHW/peer counselor home visits and mother/peer groups for improving child nutrition outcomes in LMICs. These syntheses comprise a subset of results from a systematic review that examined various nutrition intervention delivery platforms in LMICs. Our findings reveal that one-to-one home visits by CHWs/lay peer counselors are effective for improving breastfeeding practices, which is consistent with prior evidence. Lewin et al. [30] showed that using lay health workers resulted in a higher likelihood of EIBF (RR: 1.36; 95% CI: 1.14, 1.61) and EBF (RR: 2.78; 95% CI: 1.74, 4·44), when compared to care as usual. Other reviews also concluded that peer support, in one-to-one or group settings, is effective for improving EBF practices [31,32,33]. The lack of effect of the mother/peer group platform on breastfeeding in our study was, therefore, unexpected and may have been due to differences in programme factors across studies, e.g., the frequency, content, and facilitation of group sessions. We were not able to separate the effects of using trained CHWs versus peer volunteer facilitators for conducting group sessions. Further research should explore whether facilitator characteristics may affect programme targets. Further, the higher likelihood of EIBF and EBF in pooled analyses of studies involving both home visit and mother/peer group platforms suggests home visits are important for breastfeeding promotion. We speculate that this is due to the one-to-one reinforcement of knowledge and skills acquired in group sessions and/or mothers feeling more comfortable discussing and practicing breastfeeding in the privacy of home settings. Household service delivery is emphasized in the 2013 *Lancet* nutrition series [4] as a means for increasing the coverage of nutrition intervention at the community level.

However, our results suggest that the mother/peer group platform is effective for improving the quantity and quality of young children’s diets. This is likely due, in part, to participatory cooking demonstrations that frequently occur alongside counseling/coaching during group sessions and which have been shown to be effective for improving complementary feeding practices [34]. Our findings are consistent with a review of 36 studies that showed that women’s groups focused on behaviour change are beneficial for improving child feeding practices in South Asia [13].

We focused on studies [35,36,37,38,39,40,41,42,43,44,45,46,47,48,49,50,51,52,53,54,55,56,57,58,59,60,61,62,63,64,65,66,67,68,69,70,71,72,73,74,75,76,77,78,79,80,81,82,83,84,85,86,87,88,89,90] involving CHW/lay counselor home visits and or mother/peer groups intended to improve the nutrition of young children. The absence of an observed effect of either platform on child stunting and underweight in our review was not unexpected, as effects of behavioural interventions on anthropometric outcomes are generally small, due to insufficient time periods to affect changes in child growth. Moreover, as intervention–impact pathways are influenced by a complex interplay of factors at all levels, demonstrating an attributable impact of behaviour-focused interventions on nutritional status is often challenging. Nonetheless, the observed 23% reduction in the odds of <5 wasting using the combined home visit and group platform is encouraging, and suggests acute thinness may be more responsive to education/behaviour change interventions than the long-term nutritional deficits underlying stunting.

The moderate to high study heterogeneity in our review was expected. Though too few studies prevented subgroup investigations of sources of heterogeneity, we speculate much of the heterogeneity is attributable to underlying differences in implementation-related factors affecting coverage levels achieved. Differences in coverage varied by platform, with home visits achieving higher average coverage than peer groups (59.5% vs. 50.5%) among included studies. In a study in Bangladesh, Owais et al. [86] reported that 83% of mothers received a CHW home visit within the previous month, while only 13% reported attending a mother-to-mother support group for nutrition education. In a Lady Health Worker study in Pakistan, Yousafzai et al. [90] reported that 75% of households received monthly home visits, while 31% of caregivers participated in monthly group meetings. Lastly, Nair et al. [85] reported that 80% of mothers received a home visit and 56% of mothers attended a group meeting during the previous three months, in a study examining the impact of women’s groups and home-based counseling on child growth in Jharkhand and Odisha, India.

Differences in study populations, CHW/lay counselor training/performance, exposure intensity (frequency of home visits and groups), and intervention duration also likely affected the coverage rates achieved. We were not able to assess the effects of length of intervention exposure due to the intervention period not being clearly specified in many studies. Dose–response analyses examining outcomes in relation to intervention duration and/or coverage would be useful and should be considered in future reviews. 

All included studies in our review utilized an experimental design, though the overall quality of evidence was low for the majority of meta-analysed outcomes. A key limitation of our review was our inability to perform subgroup analyses to further investigate the high degree of heterogeneity, due to an insufficient number of studies. Though the inclusion of non-experimental studies in future reviews would generate a larger body of evidence, the introduction of additional biases would need to be weighed. Despite concerted efforts to select similar age/follow-up periods for anthropometric outcomes, variability across studies may have introduced some bias in the pooled analyses. However, this was not a concern for the infant and young child feeding (IYCF) outcomes, as time periods for ascertaining breastfeeding (0–6 months) and complementary feeding (6–23 months) practices were consistent across studies.

Our review strengthens the evidence through quantifying the effects of community home visits and peer groups and highlighting their potential value as platforms for improving IYCF practices in LMICs. As these tend to be established platforms within community-based child survival programmes in many countries, leveraging them for integrating nutrition-focused interventions that can be effectively provided by CHWs and through peer health promotion groups offers sizeable opportunities for nutrition behaviour change in these settings.

Therefore, the findings present an evidence-based rationale for continued investments in these community initiatives. Future research should account for platform-specific barriers affecting coverage to more broadly evaluate their effectiveness. 

## 5. Conclusions

Knowledge of effective delivery modalities is needed to achieve maximum gains from evidence-supported interventions. In a landscape of multiple platforms and options, our findings indicate that community home visits and peer groups are important for improving nutrition-related behaviours across diverse contexts.

## Figures and Tables

**Table 1:** Summary of outcomes for community healthcare worker (CHW)/peer counselor home visit platform.

Outcomes	Anticipated Absolute Effects		Relative Effect (95% CI)	№ of Participants (Studies)	Certainty of the Evidence (GRADE)
	Risk With Care as Usual	Risk With Home Visits			
Early initiation of breastfeeding RCTs ^a^	390 per 1000	490 per 1000	OR 1.50 (1.12 to 1.99)	23,044 (10)	⨁⨁◯◯ LOW
Early initiation of breastfeeding non RCTs ^a^	464 per 1000	609 per 1000	OR 1.80 (1.17 to 2.78)	9816 (4)	⨁⨁◯◯ LOW
Exclusive breastfeeding RCTs ^b^	350 per 1000	704 per 1000	OR 4.42 (2.28 to 8.56)	6046 (9)	⨁⨁◯◯ LOW
Exclusive breastfeeding non RCTs ^b^	421 per 1000	768 per 1000	OR 4.57 (1.36 to 15.36)	2532 (3)	⨁⨁◯◯ LOW
Stunting ^c^	431 per 1000	616 per 1000	OR 2.12 (0.46 to 9.74)	4436 (3)	⨁◯◯◯ VERY LOW
Underweight ^d^	379 per 1000	362 per 1000	OR 0.93 (0.82 to 1.06)	4515 (4)	⨁⨁◯◯ LOW

^a^ Child is breastfed within one hour of birth. ^b^ Child is provided only breastmilk up to 4–6 months of age ^c^ Height-for-age is more than two standard deviations below the WHO Child Growth Standards median. ^d^ Weight-for-age is more than two standard deviations below the WHO Child Growth Standards median.

**Table 2:** Summary of outcomes for mother/peer group platform.

Outcomes	Anticipated Absolute Effects		Relative Effect (95% CI)	№ of Participants (Studies)	Certainty of the Evidence (GRADE)
	Risk with Care as Usual	Risk with Group Sessions			
Early initiation of breastfeeding ^a^	651 per 1000	672 per 1000	OR 1.10 (0.92 to 1.32)	28,111 (5)	⨁⨁⨁◯ MODERATE
Exclusive breastfeeding ^b^	413 per 1000	556 per 1000	OR 1.78 (0.93 to 3.41)	2900 (4)	⨁⨁⨁◯ MODERATE
Minimum dietary diversity ^c^	346 per 1000	554 per 1000	OR 2.34 (1.17 to 4.70)	3701 (4)	⨁⨁◯◯ LOW
Minimum meal frequency ^d^	808 per 1000	907 per 1000	OR 2.31 (1.61 to 3.31)	2946 (3)	⨁⨁⨁⨁ HIGH
Stunting ^e^	352 per 1000	315 per 1000	OR 0.85 (0.68 to 1.07)	6077 (3)	⨁⨁⨁◯ MODERATE
Underweight ^f^	382 per 1000	319 per 1000	OR 0.76 (0.53 to 1.07)	6019 (3)	⨁⨁◯◯ LOW
Wasting ^g^	231 per 1000	206 per 1000	OR 0.86 (0.65 to 1.14)	5924 (3)	⨁⨁◯◯ LOW

^a^ Child is breastfed within one hour of birth. ^b^ Child is provided only breastmilk up to 4–6 months of age ^c^ Child aged 6–23 months is fed a daily minimum of four out of seven food groups according to WHO guidelines [25]. ^d^ Child aged 6–23 months is provided the appropriate number of daily solid/semi-solid/milk feeds (based on age and breastfeeding status) according to WHO guidelines [25]. ^e^ Height-for-age is more than two standard deviations below the WHO Child Growth Standards median. ^f^ Weight-for-age is more than two standard deviations below the WHO Child Growth Standards median. ^g^ Weight-for-height is more than two standard deviations below the WHO Child Growth Standards median.

**Table 3:** Summary of outcomes for using both CHW/peer counselor home visit and mother/peer group platforms.

Outcomes	Anticipated Absolute Effects		Relative Effect (95% CI)	№ of Participants (Studies)	Certainty of the Evidence (GRADE)
	Risk with Care as Usual	Risk with Home Visits and Group Sessions			
Early initiation of breastfeeding ^a^	443 per 1000	628 per 1000	OR 2.13 (1.12 to 4.05)	23,786 (9)	⨁◯◯◯ VERY LOW
Exclusive breastfeeding ^b^	302 per 1000	513 per 1000	OR 2.43 (1.70 to 3.46)	26,013 (12)	⨁◯◯◯ VERY LOW
Stunting ^c^	315 per 1000	309 per 1000	OR 0.97 (0.72 to 1.30)	23,000 (8)	⨁⨁◯◯ LOW
Underweight ^d^	146 per 1000	125 per 1000	OR 0.84 (0.61 to 1.14)	19,108 (6)	⨁⨁◯◯ LOW
Wasting ^e^	59 per 1000	46 per 1000	OR 0.77 (0.67 to 0.89)	17,610 (4)	⨁⨁◯◯ LOW

^a^ Child is breastfed within one hour of birth. ^b^ Child is provided only breastmilk up to 4–6 months of age ^c^ Height-for-age is more than two standard deviations below the WHO Child Growth Standards median. ^d^ Weight-for-age is more than two standard deviations below the WHO Child Growth Standards median. ^e^ Weight-for-height is more than two standard deviations below the WHO Child Growth Standards median.

## Supplementary Materials

The following are available online at https://www.mdpi.com/2072-6643/12/2/440/s1, Table S1: MEDLINE search strategy. Ovid MEDLINE(R) Epub Ahead of Print, In-Process & Other Non-Indexed Citations, Ovid MEDLINE(R) Daily and Ovid MEDLINE(R) 1946 to Present, Table S2: Characteristics of included studies for home visit and group platforms.
