# Supplementary material for: The Effects of Community Home Visit and Peer Group Nutrition Intervention Delivery Platforms on Nutrition Outcomes in Low and Middle-Income Countries: A Systematic Review and Meta-Analysis
Source: Nutrients. 2020 Feb 10;12(2):440. doi: 10.3390/nu12020440 (PMC7071285; doi:10.3390/nu12020440)
Supplement: Supplementary file 1 [file nutrients-12-00440-s001.pdf]

nutrients

MDPI

Table S1. MEDLINE search strategy. Ovid MEDLINE(R) Epub Ahead of Print, In-Process & Other Non-Indexed Citations, Ovid MEDLINE(R) Daily and Ovid MEDLINE(R) 1946 to Present.

#	Searches	Results
1	(pediatric* or paediatric* or child* or newborn* or congenital* or infan* or baby or babies or neonat* or "pre-term" or preterm* or "premature birth*" or NICU or preschool* or "pre-school*" or kindergarten* or kindergarden* or "elementary school*" or "nursery school*" or ("day care*" not adult*) or schoolchild* or toddler* or boy or boys or girl* or "middle school*" or pubescen* or juvenile* or teen* or youth* or "high school*" or adolesc* or "pre-pubesc*" or prepubesc*).tw,kf. or (child* or adolesc* or pediat* or paediat*).jn.	2419824
2	Prenatal care/ or Perinatal care/ or obstetrics/ or breast feeding/	80549
3	women/ or pregnant women/	20520
4	(obstetric* or gynecolog* or gynaecolog* or perinatal or prenatal or "pre natal" or antenatal or "ante natal" or postnatal or "post natal" or "maternal health" or gestation or pregnancies or pregnant or pregnancy or childbearing or gravidity or mother* or breastfeed* or "breast feeding" or woman or women).tw,kf.	1659718
5	or/1-4	3675093
6	Developing Countries.sh,kf.	80569
7	(Africa or Asia or Caribbean or "West Indies" or "South America" or "Latin America" or "Central America").tw,kf,hw,cp.	238412
8	(Afghanistan or Albania or Algeria or Angola or Antigua or Barbuda or Argentina or Armenia or Armenian or Aruba or Azerbaijan or Bahrain or Bangladesh or Barbados or Benin or Byelarus or Byelorussian or Belarus or Belorussian or Belorussia or Belize or Bhutan or Bolivia or Bosnia or Herzegovina or Hercegovina or Botswana or Brasil or Brazil or Bulgaria or "Burkina Faso" or "Burkina Fasso" or "Upper Volta" or Burundi or Urundi or Cambodia or "Khmer Republic" or Kampuchea or Cameroon or Cameroons or Cameron or Camerons or "Cape Verde" or "Central African Republic" or Chad or Chile or China or Colombia or Comoros or "Comoro Islands" or Comores or Mayotte or Congo or Zaire or "Costa Rica" or "Cote d'Ivoire" or "Ivory Coast" or Croatia or Cuba or Cyprus or Czechoslovakia or "Czech Republic" or Slovakia or "Slovak Republic" or Djibouti or "French Somaliland" or Dominica or "Dominican Republic" or "East Timor" or "East Timur" or "Timor Leste" or Ecuador or Egypt or "United Arab Republic" or "El Salvador" or Eritrea or Estonia or Ethiopia or Fiji or Gabon or "Gabonese Republic" or Gambia or Gaza or "Georgia Republic" or "Georgian Republic" or Ghana or "Gold Coast" or Greece or Grenada or Guatemala or Guinea or Guam or Guiana or Guyana or Haiti or Honduras or Hungary or India or Maldives or Indonesia or Iran or Iraq or "Isle of Man" or Jamaica or Jordan or Kazakhstan or Kazakh or Kenya or Kiribati or Korea or Kosovo or Kyrgyzstan or Kirghizia or "Kyrgyz Republic" or Kirghiz or Kirgizstan or "Lao PDR" or Laos or Latvia or Lebanon or Lesotho	3296449

	or Basutoland or Liberia or Libya or Lithuania or Macedonia or Madagascar or "Malagasy Republic" or Malaysia or Malaya or Malay or Sabah or Sarawak or Malawi or Nyasaland or Mali or Malta or "Marshall Islands" or Mauritania or Mauritius or "Agalega Islands" or Mexico or Micronesia or "Middle East" or Moldova or Moldovia or Moldovian or Mongolia or Montenegro or Morocco or Ifni or Mozambique or Myanmar or Myanma or Burma or Namibia or Nepal or "Netherlands Antilles" or "New Caledonia" or Nicaragua or Niger or Nigeria or "Northern Mariana Islands" or Oman or Muscat or Pakistan or Palau or Palestine or Panama or Paraguay or Peru or Philippines or Philipines or Phillipines or Phillippines or Poland or Portugal or Puerto Rico or Romania or Rumania or Roumania or Russia or Russian or Rwanda or Ruanda or "Saint Kitts" or "St Kitts" or Nevis or "Saint Lucia" or "St Lucia" or "Saint Vincent" or "St Vincent" or Grenadines or Samoa or "Samoan Islands" or "Navigator Island" or "Navigator Islands" or "Sao Tome" or "Saudi Arabia" or Senegal or Serbia or Montenegro or Seychelles or "Sierra Leone" or Slovenia or "Sri Lanka" or Ceylon or "Solomon Islands" or Somalia or "South Africa" or Sudan or Suriname or Surinam or Swaziland or Syria or Tajikistan or Tadzhikistan or Tadjikistan or Tadzhik or Tanzania or Thailand or Togo or "Togolese Republic" or Tonga or Trinidad or Tobago or Tunisia or Turkey or Turkmenistan or Turkmen or Uganda or Ukraine or Uruguay or USSR or "Soviet Union" or "Union of Soviet Socialist Republics" or Uzbekistan or Uzbek or Vanuatu or "New Hebrides" or Venezuela or Vietnam or "Viet Nam" or "West Bank" or Yemen or Yugoslavia or Zambia or Zimbabwe or Rhodesia).hw,kf,ti,ab,cp.	
9	((developing or "less* developed" or "under developed" or underdeveloped or "middle income" or "low* income" or underserved or "under served" or deprived or poor*) adj1 (countr* or nation? or population? or world)).tw.	82577
10	((developing or "less* developed" or "under developed" or underdeveloped or "middle income" or "low* income") adj1 (economy or economies)).tw.	431
11	(low* adj1 (gdp or gnp or "gross domestic" or "gross national")).tw.	214
12	(low adj3 middle adj3 countr*).tw.	10025
13	(lmic or lmics or "third world" or "lami countr*").tw.	5386
14	"transitional countr*".tw.	142
15	or/6-14	3431580
16	5 and 15	638493
17	exp mass media/ or exp social media/ or blogging/ or social networking/ or radio/	49544
18	(internet or "cyber space" or cyberspace or "world wide web" or "information and communication technologies" or "communication technolog*" or ICTs or "communication application*" or "communication apps").tw,kf.	47947
19	((virtual or online) adj2 (communit* or network* or forum? or support*)).tw,kf.	3687

20	("mass media" or "social media" or "social medium?" or "social network*" or sns or multimedia).tw,kf.	32173
21	(phone adj2 (app? or application?)).tw,kf.	654
22	("push technolog*" or "web 2.0" or "web 2.0s" or radio or television* or TV or TVs or twitter or facebook or WeChat or weibo or YouTube or whatsapp or blogging or blog? or "hash tag*" or hashtag* or microblog* or "chat room" or "internet chat" or "online chat" or email or wiki*).tw,kf.	71957
23	(sms or "short messag*" or text* or "mobile outreach" or "mobile out reach" or mms or smartphone* or telephone* or cellphone*).tw,kf.	186576
24	((text or short or instant or multimedia or "multi-media") adj1 messag*).tw,kf.	3561
25	((car or cell or cellular or mobile or smart) adj1 (phone or phones or telephone*)).tw,kf.	9888
26	(mobile adj2 communication).tw,kf.	553
27	(mhealth or "m-health" or ehealth or "e-health" or "mobile medicine" or "mobile health").tw,kf.	7698
28	or/17-27	363999
29	16 and 28	12150
30	((cash or money or monetary or electronic) adj1 (transfer* or assistant* or voucher* or allowance?)).tw,kf.	856
31	("mobile money" or "mobile pay" or "e-transfer" or "etranfer" or "financial assistance" or "financial support" or "food stamp?" or "food voucher?" or "food assistance" or "cash credit").tw,kf.	5313
32	food supply/ or ("food insecurity*" or "food security" or "food suppl*" or "social protection").tw,kf.	21224
33	or/30-32	26978
34	16 and 33	3637
35	("community health" adj1 (service? or centre? or center? or promotion or "out reach" or outreach)).tw,kf.	5192
36	(("health care" or healthcare or health) adj1 (worker? or personnel or volunteer* or provider* or network or campaign* or alliance)).tw,kf.	55879
37	((community or "community based") adj1 (program* or network* or mobili?er or "out reach" or outreach or aide? or involvement or participation)).tw,kf.	7874
38	("volunt* worker?" or "peer group?" or "wom#n group?" or "wom#n's group?" or "child health day" or "integrated management of childhood illness" or	5088

	IMCI).tw,kf.	
39	or/35-38	72293
40	16 and 39	12726
41	29 or 34 or 40	27224
42	("macro-nutrient" or "macro nutrient" or macronutrient or malnutrition or malnourishment or nutraceutical* or undernutrition or undernourishment or underfeeding or malabsorption or nourish*).tw,kf.	66558
43	(diet? or dietary or nutrition* or supplement* or food? or feed*).tw,kf.	1345156
44	or/42-43	1373414
45	41 and 44	6738
46	limit 45 to yr="1997 -Current"	4814
47	limit 46 to english language	4428

nutrients

MDPI

Table S2: Characteristics of included studies for home visit and group platforms [a, b]

Author	Year	Country	Study / trial / intervention period	Study design	Participants	Study sample	Platform	Intervention	Outcomes
Ara et al [35]	2018	Bangladesh	Enrolled women between September 2014 and July 2016	Cluster-randomized trial	Mother-child dyads	350	Home visits	CHW made 10 home counseling visits for prenatal care, nutrition and IYCF practices: 3 in the last trimester of pregnancy, 3 in the 1st month after delivery (1 within 48 hr of delivery, 1 10–14 days after, and 1 24–28 days after), and 4 visits during 2 to 6 months after delivery	EIBF<br>EBF<br>LBW<br>Birth weight
Aracena et al [36]	2009	Chile	15 months	Randomized controlled trial	Adolescent mother-child dyads	90	Home visits	CHW home visits from 3rd trimester to 1 year of age for maternal and child health and nutrition counseling	Anemia<br>Underweight
Attanasio et al [37]	2014	Colombia	Intervention period February-May 2010 to September-December 2011; intervention 18 months	Cluster-randomized trial	Mother-child dyads	626	Home visits	CHW home visits for education and provision of multiple micronutrient powder supplements	Hemoglobin<br>Height<br>Weight
Ayiasi et al [38]	2016	Uganda	Study period May/June 2013 to October/December 2014	Randomized controlled trial	Mother-infant dyads	1385	Home visits	CHW prenatal and postnatal home visits for maternal and newborn care counseling	EIBF

Balaluka et al [39]	2012	DRC	Project period 2004 to 2006	Quasi-experimental evaluation	Mother-child dyads	386	Home visits	CHW promotion of exclusive breastfeeding from birth through door-to-door visits	EBF Weight
Gabida et al [40]	2015	Zimbabwe	Intervention period 3 months	Two-by-two factorial cluster randomized trial	Mother-child dyads	357	Home visits	CHW provided breastfeeding education at facility or community	EIBF EBF
Haider et al [41]	2000	Bangladesh	Enrolment February - December 1996; infants followed to 5 months of age	Cluster-randomized trial	Mother-child dyads	726	Home visits	CHW home visits: 2 in last trimester of pregnancy, 4 in first month (1 within 48 h of delivery, 1 on day 5, 1 during days 10–14, and 1 during days 24–28); Fortnightly visits in months 2–5; Total number of scheduled visits was 15 for breastfeeding promotion	EIBF EBF
Hanson et al [42]	2015	Tanzania	Study period 2010-2013	Cluster-randomized trial	Mother-child dyads	14,295	Home visits	CHW home-based counselling for maternal care and early and exclusive breastfeeding 3 times in pregnancy and 2 times in early postpartum.	EIBF
Ijumba et al [43]	2015	South Africa	Participants recruited June 2008 to December 2010 and data collection was concluded July 2011	Cluster-randomized trial	Mother-infant dyads	3494	Home visits	CHW home visits during pregnancy and postpartum for infant feeding counseling	EIBF

Kimani-Murage et al [44]	2017	Kenya	Recruitment September 2012 to February 2014	Cluster-randomized trial	Mother-child dyads	1110	Home visits	CHW home visits for maternal, infant and young child nutrition counseling	EBF
Leite et al [45]	2005	Brazil	Trial period November 1996 to April 1997	Randomized controlled trial	Mother-newborn dyads	1003	Home visits	CHW home visits from postpartum to 120 days after birth for breastfeeding counseling	EBF
McDougal et al [46]	2017	India	Baseline January-April 2012; Endline January-April 2014	Quasi-experimental study	Mother-child dyads	7191 Baseline; 6143 Endline (cross-sectional)	Home visits	Accredited Social Health Activist, auxiliary nurse midwife, and Anganwadi worker home visits in pregnancy and early postpartum for RMNH counseling	EIBF
Nayak et al [47]	2016	India	Study period September 2006 to July 2009	Cluster-randomized trial	Mother-child dyads	77	Home visits	Home visits for nutrition and IYCF counseling starting during the third trimester of pregnancy and after delivery at 2, 5, 8, 11, 14, 16 and 20 months of age	EIBF<br>EBF<br>Continued BF<br>Birth weight<br>Length<br>Weight
Nguyen et al [48]	2017	Bangladesh	Baseline 2015; Endline 2016	Cluster-randomized evaluation	Mother-infant dyads	Baseline 2000; Endline 2000	Home visits	Health worker and CHW home visits during pregnancy and postpartum for maternal and infant care counseling	MDD<br>EIBF<br>EBF
Nguyen et al [49]	2018	Bangladesh	Baseline June-Aug 2015; Endline June- Aug	Cluster-randomized evaluation	Pregnant and post- partum mothers	Baseline 2000; Endline 2000 (cross	Home visits	Health worker and CHW home visits during pregnancy and postpartum for maternal and infant care counseling	IFA coverage

			2016			sectional)			
Ochola et al [50]	2013	Kenya	Trial period April 2006 to April 2008	Cluster-randomized trial	Mother-child dyads	240	Home visits	7 home visit counseling sessions: 1 prenatal, 1 during the first week after delivery, and the 3rd to 7th monthly up to 5 months postpartum	EBF
Penfold et al [51]	2014	Tanzania	Study period 2010-2011	Cluster-rando mized trial	Mother-child dyads	512	Home visits	CHW home visits during pregnancy and postpartum for newborn care and breastfeeding counseling	EIBF
Rahman et al [52]	2016	Bangladesh	Baseline 2008; Endline 2012	Quasi-experimental evaluation	Mother-child dyads	Baseline 3118; Endline 1527	Home visits	CHW home visits for maternal newborn, child health and nutrition counseling	EIBF
Risonar et al [53]	2008	Philippines	Baseline June 2002; Endline January 2003	Controlled before- after study	Pregnant women	Baseline 598; Endline 582	Home visits	CHW/TBA home visit prenatal care counseling and IFA distribution	IFA consumption<br>Anemia
Saleem et al [54]	2014	Pakistan	Intervention 30 weeks	Cluster-rando mized trial	Mother-child dyads	212	Home visits	CHW home visits for IYCF counseling	Stunting<br>Underweight<br>Wasting
Singh et al (a) [55]	2017	India	Study period 2004-2006	Quasi-experi mental study	Mother-child dyads	957	Home visits	Anganwadi workers counseled on breastfeeding and complementary feeding	EIBF<br>EBF<br>Stunting<br>Underweight<br>LAZ<br>WAZ

									Length<br>Weight
Singh et al (b) [56]	2017	India	Study period May 2004 to July 2006	Quasi-experimental evaluation	Mother-child dyads	942	Home visits	Anganwadi worker home visits from pregnancy to 2 years for IYCF counseling	EIBF<br>EBF<br>VAS
Vir et al [57]	2014	India	Project period 2009 - 2011	Quasi-experimental study	Mother-child dyads	3626	Home visits	CHW home visits for IYCF counseling	EIBF<br>EBF<br>Underweight<br>Stunting<br>Wasting<br>IFA consumption
Zhou et al [58]	2016	China	Baseline April and October 2013; Endline October 2013 and April 2014 ; intervention period 6 months	Cluster-randomized trial	Mother-child dyads	1818	Home visits<br><br>Multi-platform	Intervention 1: One-on-one counseling on nutrition and feeding practices and 6-month supply of MNP<br><br>Intervention 2: One-on-one counseling on nutrition and feeding practices and 6-month supply of MNP plus daily text MNP reminder messages	Anemia<br>MNP consumption
Fottrell et al [59]	2013	Bangladesh	Trial period January 2009 to June 2011	Cluster-randomized trial	Mother-child dyads	9895	Group sessions	Monthly women's groups facilitated by CHWs relating to women's health	EIBF
Harris-Fry et al	2016	Bangladesh	Baseline survey	Controlled	WRA	Baseline 5355;	Group	Monthly women's groups for health/nutrition	Women's dietary

[60]			October-December 2011; Endline survey February-March 2013; intervention lasted 13 months	before-after study		Endline 5128	sessions	promotion	diversity
Kang et al [61]	2017	Ethiopia	Intervention period August 2012 to August 2013	Cluster-randomized trial	Mother-child dyads	1790	Group sessions	CHW led 2 week daily group nutrition education and cooking session and child growth monitoring	Dietary diversity
Kang et al (b) [62]	2017	Ethiopia	Intervention period August 2012 to August 2013	Evaluation using cluster-randomized trial data	Mother-child dyads	1790	Group sessions	CHW led 2 week daily group nutrition education and cooking session and child growth monitoring	Continued BF MDD MMF
Kang et al (a) [63]	2017	Ethiopia	Intervention period August 2012 to August 2013	Cluster-randomized trial	Mother- child dyads	1790	Group sessions	CHW led 2 week daily group nutrition education and cooking session and child growth monitoring	LAZ WAZ WHZ Stunting Wasting Underweight
Kuchenbecker et al [64]	2017	Malawi	Baseline August/September 2011; Endline August/September 2014; intervention	Cluster-randomized trial	Mother-child dyads	Baseline 832; Endline 959	Group sessions	CHW led group sessions for IYCF promotion	WAZ HAZ WHZ MDD MMF

			period 5 months						MAD<br>Continued BF
Lewycka et al [65]	2013	Malawi	Study period Feb 2006 to Jan 2009 for the women's group intervention; Study period July 2005 to June 2008 for the volunteer peer counselling intervention	Cluster-randomized trial	Mother-child dyads	3002	Group sessions<br><br>Home visits	20 women's group meetings related to maternal and child health<br>CHW made 5 home visits: in the 3rd trimester, in the week after birth, and at 1 month, 3 months, and 5 months after delivery for health education including exclusive breastfeeding and infant care	EIBF<br>EBF
More et al [66]	2012	India	Trial period October 2006 to September 2009	Cluster-randomized trial	Mothers	15,192	Group sessions	CHW led women's group sessions for prenatal care promotion	IFA consumption
Mushaphi et al [67]	2015	South Africa	Study period 2007-2009; intervention period 12 months	Controlled before- after study	Mother-child dyads	Baseline 129; Endline 89	Group sessions	Group nutrition education sessions twice a week on child feeding	Stunting<br>Underweight<br>Wasting
Negash et al [68]	2014	Ethiopia	Study period September 2012 to March 2013	Controlled before- after study	Mother-child dyads	Baseline 197; Endline 153	Group sessions	Group sessions for mothers on IYCF promotion including cooking demonstration	Continued BF<br>MMF<br>MDD

Tripathy et al [69]	2016	India	Baseline Sept 2009 to Aug 2010; Endline Jan 2011 to Dec 2012	Cluster-randomized trial	Mother-child dyads	7219	Group sessions	Accredited Social Health Activists led women's group meetings during pregnancy for maternal and newborn health promotion	EIBF
Yadav et al [70]	2014	Nepal	Study period February 2012 to November 2013	Controlled before- after study	Mother-child dyads	Baseline 615; Endline 708	Group sessions	CHW led monthly nutrition education group sessions	Stunting<br>Underweight<br>Wasting<br>EIBF<br>EBF<br>VAS
Younes et al [71]	2015	Bangladesh	Intervention period 2010-2011	Controlled before-after study	Mother-child dyads	Baseline 1897; Endline 2270	Group sessions	Women's groups for maternal, neonatal and child health promotion	EBF<br>MDD
Saville et al [72]	2018	Nepal	Project period 2013 to 2015	Cluster-randomized trial	Mother-infant dyads	7079	Group sessions<br><br>Multi-platform	Intervention 1: CHW led monthly women's groups for prenatal health and nutrition promotion<br><br>Intervention 2: CHW led monthly women's groups for prenatal health and nutrition promotion plus cash transfer (US$ 7.5/month)	WAZ<br>HAZ<br>WHZ<br>EIBF<br>EBF<br>Continued BF<br>Dietary diversity<br>Birth weight<br>LBW<br>Underweight<br>Stunting<br>Wasting

Bhandari et al [73]	2003	India	Infants born between October 1999 and June 2000 followed to 6 months of age	Cluster-randomized trial	Mother-child dyads	1025	Home visits and group sessions	Monthly home visits for new mothers until child aged 12 months including IYCF messages and weighing once every 3 months for children 2 yrs old conducted by Anganwadi workers; monthly meetings conducted by the auxiliary nurse midwives with community representatives who held neighborhood meetings once a month with caretakers of children 2 yrs old including nutrition counseling	EBF<br>Wasting<br>Stunting<br>Weight<br>Length
Bhandari et al [74]	2004	India	Infants born between October 1999 and June 2000 followed to 18 months of age	Cluster-randomized trial	Mother-child dyads	1025	Home visits and group sessions	Monthly home visits for new mothers until child aged 12 months including IYCF messages and weighing once every 3 months for children 2 yrs old conducted by Anganwadi workers; monthly meetings conducted by the auxiliary nurse midwives with community representatives who held neighborhood meetings once a month with caretakers of children 2 yrs old including nutrition counseling	Underweight<br>Weight<br>Stunting<br>Length
Brasington et al [75]	2016	Egypt	Baseline September-October 2012; Endline January-February 2014	Quasi-experimental e valuation	Mother-child dyads	Baseline 3199; Endline 3445	Home visits and group sessions	CHW led group sessions with pregnant women, home visits for postpartum women, group sessions for mothers of children 6 months to 2 years and growth monitoring	IFA consumption<br>EBF

Brenner et al [76]	2011	Uganda	Project period 2006-2009	Controlled before- after evaluation	Mother-child dyads	Baseline 1118; Endline 1092	Home visits and group sessions	CHW home visits and group sessions for child health/nutrition promotion	Underweight
Bhutta et al [77]	2008	Pakistan	Baseline May - June 2003; Endline August -September 2005	Quasi-experimental study	Mother-child dyads	770	Home visits and group sessions	Lady Health Worker home visits during pregnancy and postpartum and group education sessions for prenatal and neonatal care, breastfeeding promotion	EIBF<br>EBF
Crookston et al [78]	2007	Cambodia	Baseline 2004; Endline 2005 (1 year later)	Controlled before- after study	Mother-child dyads	Baseline 440; Endline 467	Home visits and group sessions	Community "nun" home visits and group sessions for breastfeeding counseling	EIBF<br>EBF
Fenn et al [79]	2012	Ethiopia	Baseline 2004; Endline 2009	Controlled before- after evaluation	Mother-child dyads	Baseline 2509; Endline 1719	Home visits and group sessions	CHW home visits and group education sessions on maternal nutrition and IYCF practices	HAZ<br>Stunting<br>Dietary diversity<br>VAS
Kung'u et al [80]	2018	Ethiopia, Kenya, Senegal	Baseline 2013; Endline 2015	Quasi-experimental evaluation	Mother-child dyads	Ethiopia: Baseline 1968; Endline 1460<br><br>Kenya: Baseline 682;	Home visits and group sessions	Home and group behaviour change counseling for maternal and child health and nutrition	IFA consumption<br>EIBF<br>EBF

						Endline 735<br><br>Senegal: Baseline 1925; Endline 1995			
Lamstein et al [81]	2017	Nigeria	Baseline December 2014 to June 2015; Endline January and March 2017; 18 months of implementation	Quasi-experimental evaluation	Mother-child dyads	Baseline 3525; Endline 5366	Home visits and group sessions	CHW led group meetings, home visits, and community events for maternal, infant, and young child health and nutrition promotion	EIBF<br>EBF<br>Continued BF<br>Stunting<br>Underweight<br>Wasting<br>MMF<br>MAD<br>MDD
Liang et al [82]	2018	China	Baseline 2001; Endline 2005	Controlled before- after study	Mother-child dyads	Baseline 11,704; Endline 11118	Home visits and group sessions	CHW health and nutrition counseling, child growth monitoring	EIBF<br>EBF<br>Stunting<br>Underweight<br>Wasting<br>VAS
Mayhew et al [83]	2014	Afghanistan	Matched comparison analysis between June and August	Pre-post evaluation	Mother-child dyads	828	Home visits and group sessions	CHW nutrition counseling and growth monitoring	WAZ

			2011						
Memon et al [84]	2015	Pakistan	Intervention period 18 months	Quasi-experimental evaluation	Mother-child dyads	Baseline 708; Endline 677	Home visits and group sessions	Lady Health Worker/CHW home visits, group education/video sessions for maternal and newborn health and nutrition promotion	EIBF
Nair et al [85]	2017	India	Study included infants born to pregnant women recruited between Oct 2013 and Feb 2015	Cluster-randomized trial	Mother-child dyads	3001	Home visits and group sessions	CHW home visits from pregnancy to 2 years on IYCF; monthly women's group meetings to promote nutrition	Birthweight<br>Stunting<br>Wasting<br>Underweight<br>LAZ<br>WHZ<br>WAZ<br>EBF<br>MDD<br>MMF
Owais et al [86]	2017	Bangladesh	Project period January 2011 to January 2014	Quasi-experimental study	Mother- child dyads	2400	Home visits and group sessions	CHW home visits for IYCF counseling; mothers support groups	Stunting<br>LAZ<br>WLZ<br>MAD
Rockers et al [87]	2016	Zambia	Baseline August/September 2014; Endline September/October	Cluster-randomized trial	Mother-child dyads	526	Home visits and group sessions	CHW home visits for child education and malnutrition screening; mother group meetings for child health and nutrition promotion	HAZ<br>Stunting<br>WAZ<br>Underweight

			2015; 1-year study intervention period						Dietary diversity
Shi et al [88]	2009	China	Baseline April–September 2006 (2–4 months of age); followed to 12 months of age	Cluster-randomized trial	Mother-child dyads	599	Home visits and group sessions	Group sessions and home visits for IYCF counseling and recipes	Continued BF<br>Meal frequency<br>Weight<br>Length
Walsh et al [89]	2002	South Africa	Project period 2 years	Quasi-experimental evaluation	Mother-child dyads	Baseline 536; Endline 815	Home visits and group sessions	CHW home visits and community groups for nutrition promotion	Wasting<br>Stunting<br>Underweight
Yousafzai et al [90]	2014	Pakistan	Trial period between June 2009 to March 2012	Cluster-randomized trial	Mother-child dyads	732	Home visits and group sessions	Lady Health Worker home visits and group meetings for nutrition education; children 6–24 months given MNP supplement	HAZ<br>WAZ<br>WHZ

nutrients

MDPI

[a] BF, breastfeeding; EIBF, early initiation of breastfeeding; EBF, exclusive breastfeeding; IFA, iron folic acid; LBW, low birth weight; MNP, multiple micronutrient powder; MDD, minimum dietary diversity; MMF, minimum meal frequency; MAD, minimum acceptable diet; HAZ, height-for-age z-score; LAZ, length-for-age z-score; VAS, vitamin A supplementation; WAZ, weight-for-age z-score; WHZ, weight-for-height z-score. N = 82 as Yousafzai, 2018 (doi: 10.1111/nyas.13649) included only for coverage outcome.

[b] Control is care as usual unless otherwise indicated in the intervention column.
